# The SspA subtilisin-like protease of *Streptococcus suis *triggers a pro-inflammatory response in macrophages through a non-proteolytic mechanism

**DOI:** 10.1186/1471-2180-11-47

**Published:** 2011-03-01

**Authors:** Laetitia Bonifait, Daniel Grenier

**Affiliations:** 1 Groupe de Recherche en Écologie Buccale (GREB), Faculté de médecine dentaire, Université Laval, Quebec City, Quebec, Canada; 2 Centre de Recherche en Infectiologie Porcine (CRIP), Fonds Québécois de la Recherche sur la Nature et les Technologies (FQRNT), Quebec, Canada

## Abstract

**Background:**

*Streptococcus suis *is a major swine pathogen worldwide that causes meningitis, septicemia, arthritis, and endocarditis. Using animal models, a surface-associated subtilisin-like protease (SspA) has recently been shown to be an important virulence factor for *S. suis*. In this study, we hypothesized that the *S. suis *SspA subtilisin-like protease may modulate cytokine secretion by macrophages thus contributing to the pathogenic process of meningitis.

**Results:**

Phorbol 12-myristate 13-acetate-differentiated U937 macrophages were stimulated with recombinant SspA prior to monitor cytokine secretion by ELISA. Our results indicated that the recombinant SspA was able to dose-dependently induce IL-1β, IL-6, TNF-α, CXCL8 and CCL5 secretion in macrophages. The heat-inactivated protease was still able to induce cytokine secretion suggesting a non-proteolytic mechanism of macrophage activation. Using specific kinase inhibitors, evidence were bought that cytokine secretion by macrophages stimulated with the recombinant SspA involves the mitogen-activated protein kinase signal transduction pathway. While stimulation of macrophages with low concentrations of recombinant SspA was associated to secretion of high amounts of CCL5, the use of recombinant SspA at a high concentration resulted in low amounts of CCL5 detected in the conditioned medium. This was found to be associated with a proteolytic degradation of CCL5 by SspA. The ability of SspA to induce cytokine secretion in macrophages was confirmed using a mutant of *S. suis *deficient in SspA expression.

**Conclusion:**

In conclusion, this study identified a new mechanism by which the *S. suis *SspA may promote central nervous system inflammation associated with meningitis.

## Background

*Streptococcus suis *is a major swine pathogen worldwide that causes meningitis, septicemia, arthritis, and endocarditis [1]. *S. suis *infections in humans remain sporadic and affect mainly individuals in close contact with sick or carrier pigs or pig-derived products, typically pig farmers, veterinary personnel, abattoir workers, and butchers [2]. However, the important outbreak that occurred in China in 1998 and 2005 modified the world perspective regarding the threat of *S. suis *for humans [3,4]. *S. suis *is transmitted via the respiratory route and colonizes the palatine tonsils of pigs. While 35 serotypes (1 to 34 and 1/2) have been identified, serotype 2 is considered the most frequently associated with pathology [5], although other serotypes are also the source of many infections [6-8].

Various potential virulence factors produced by *S. suis *have been identified, including a sialic acid-rich capsule [9], an hemolysin (suilysin) [10], adhesins [11,12], and proteolytic enzymes [13,14]. Our laboratory recently reported on the cloning of a 170 kDa subtilisin-like protease (SspA) found on the cell surface of *S. suis *[15]. This protease was found to possesses a high protein cleavage specificity and can degrade the Aα chain of fibrinogen thus preventing thrombin-mediated fibrin formation [15]. Using animal models and deficient-mutants, the surface-associated SspA was found to play a key role as virulence factor for *S. suis *[16,17]. However, the exact contribution of the SspA in the pathogenic process of *S. suis *infections has not been clearly defined.

To cause meningitis, *S. suis *must first cross the mucosal barrier, enter the bloodstream, resist to host defense mechanisms in the intravascular space, invade the blood-brain barrier, and then replicate in the subarachnoidal space [18]. Once the bacteria reach the blood-brain barrier, the secretion of proinflammatory cytokines, by host cells may contribute to increasing the permeability of this barrier [18-20]. A number of studies have reported that *S. suis *can induce the secretion of high amounts of proinflammatory cytokines by host cells, including monocytes/macrophages [19-21]. This excessive production of proinflammatory cytokines has been suggested to play a key role in pathogenesis of both systemic and central nervous system infections and to contribute to the pathogenic processes of meningitis [22,23]. The aim of this study was to investigate the capacity of the *S. suis *SspA subtilisin-like protease to modulate cytokine secretion by macrophages.

## Methods

### Strains and growth conditions

*S. suis *P1/7 (serotype 2) as well as a SspA deficient mutant (G6G) were used in this study. Mutant G6G was selected from a mutant library constructed using the pTV408 temperature-sensitive suicide vector to deliver the Tn917 transposon into *S. suis *P1/7 via electroporation [16]. This mutant is unable to degrade the chromogenic substrate (N-succinyl-Ala-Ala-Pro-Phe-*p*Na; Sigma-Aldrich Canada Ltd., Oakville, ON, CANADA) specific for subtilisin-like proteases and showed a single Tn917 insertion into the gene coding for the SSU0757 protein in the genome of *S. suis *P1/7 [16]. Bacteria were grown at 37°C in Todd Hewitt broth (THB; BBL Microbiology Systems, Cockeysville, MA, USA).

### Preparation of recombinant SspA of *S. suis*

The subtilisin-like protease SspA of *S. suis *was cloned, purified, and characterized in a previous study [15]. Briefly, the *SSU0757 *gene encoding the SspA was amplified and a 4,798-bp DNA fragment was obtained. It was cloned into the expression plasmid pBAD/HisB and then inserted into *Escherichia coli *to overproduce the protein. The recombinant protease was purified by chromatography procedures and showed a molecular weight of 170 kDa. Using a chromogenic *Limulus *amebocyte lysate assay (Associates of Cape Cod, Inc., East Falmouth, MA), the SspA preparation was found to contain less than 5 ng endotoxin/ml.

### Cultivation of monocytes and preparation of macrophage-like cells

The monoblastic leukemia cell line U937 (ATCC CRL-1593.2; American Type Culture Collection, Manassas, VA, USA) was cultivated at 37°C in a 5% CO_2 _atmosphere in RPMI-1640 medium (HyClone Laboratories, Logan, UT, USA) supplemented with 10% heat-inactivated fetal bovine serum (FBS; RPMI-FBS) and 100 μg/ml penicillin-streptomycin. Monocytes (2 × 10^5 ^cells/ml) were incubated in RPMI-FBS containing 10 ng/ml of phorbol 12-myristic 13-acetate (PMA) for 48 h to induce differentiation into adherent macrophage-like cells [24]. Following the PMA treatment, the medium was replaced with fresh medium and differentiated macrophages were incubated for an additional 24 h prior to use. Adherent macrophages were suspended in RPMI-FBS and centrifuged at 200 × g for 5 min. The cells were washed, suspended at a density of 1 × 10^6 ^cells/ml in RPMI supplemented with 1% heat-inactivated FBS and seeded in a 96 well-plate (1 × 10^6 ^cells/well/0.2 ml) at 37°C in 5% CO_2 _atmosphere for 2 h prior to treatments.

### Treatment of macrophages

PMA-differentiated U937 macrophages were treated with recombinant SspA at concentrations ranging from 0.00033 to 33 μg/ml. Stimulation was also performed using the recombinant SspA treated at 100°C for 30 min to inactivate the catalytic activity or in the presence of polymyxin B (1 μg/ml) to exclude any contribution of contaminating LPS in macrophage stimulation. As a control, pancreatic trypsin (Sigma-Aldrich Canada Ltd.) was used in the same range of concentrations (0.00033 to 33 μg/ml). Lastly, PMA-differentiated U937 macrophages were also stimulated with *S. suis *P1/7 and G6G cells at a multiplicity of infection (MOI) of 100. All treatments were carried out for 18 h in a 5% CO_2 _atmosphere.

### Determination of macrophage viability

Following treatments with either the recombinant SspA or bacterial cells, cell viability was evaluated with an MTT (3-[4,5-diethylthiazol-2-yl]-2,5-diphenyltetrazolium bromide) test performed according to the manufacturer's protocol (Roche Diagnostics, Mannheim, Germany).

### Determination of cytokine secretion

Commercial enzyme-linked immunosorbent assay (ELISA) kits (R&D Systems, Minneapolis, MN, USA) were used to quantify IL-1β, IL-6, TNF-α, CCL5, and CXCL8 concentrations in the cell-free culture supernatants according to the manufacturer's protocols. The absorbance at 450 nm was read using a microplate reader with the wavelength correction set at 550 nm. The rated sensitivities of the commercial ELISA kits were 3.9 pg/ml for IL-1β, 9.3 pg/ml for IL-6, 15.6 pg/ml for TNF-α and CCL5, and 31.2 pg/ml for CXCL8.

### Determination of cytokine degradation

Degradation of IL-6, CXCL8, and CCL5 by the recombinant SspA was assessed by ELISA. Briefly, recombinant cytokines (300 pg/ml of IL-6, 250 pg/ml of CXCL8, or 500 pg/ml of CCL5,) were incubated with the recombinant SspA at concentrations ranging from 0.26 to 16.5 μg/ml for 4 h. Following incubation, residual cytokines were quantified by ELISA as described above.

### Effect of kinase inhibitors on cytokine secretion

Specific kinase inhibitors (Calbiochem, Mississauga, ON, Canada) used at the optimal concentration recommended by the manufacturer (0.0625 μM) were added to macrophages 2 h prior to being treated with the recombinant SspA (0.33 μg/ml) for 18 h. The inhibitors SB203580 [p38 mitogen-activated kinase (p38MAPK) inhibitor], UO126 [mitogen-activated extracellular kinase 1, 2 (MEK 1, 2) inhibitor] and JNK inhibitor II [c-JUN N-terminal kinase (JNK) inhibitor], were evaluated for their effect on IL-6, CXCL8, and CCL5 secretion by macrophages.

### Statistical analysis

All treatments and cytokine determination were performed in triplicate and the means ± standard derivations were calculated. Differences were analyzed for statistical significance using the Student's t-test and were considered significant at P < 0.01.

## Results

**Figure 1:**
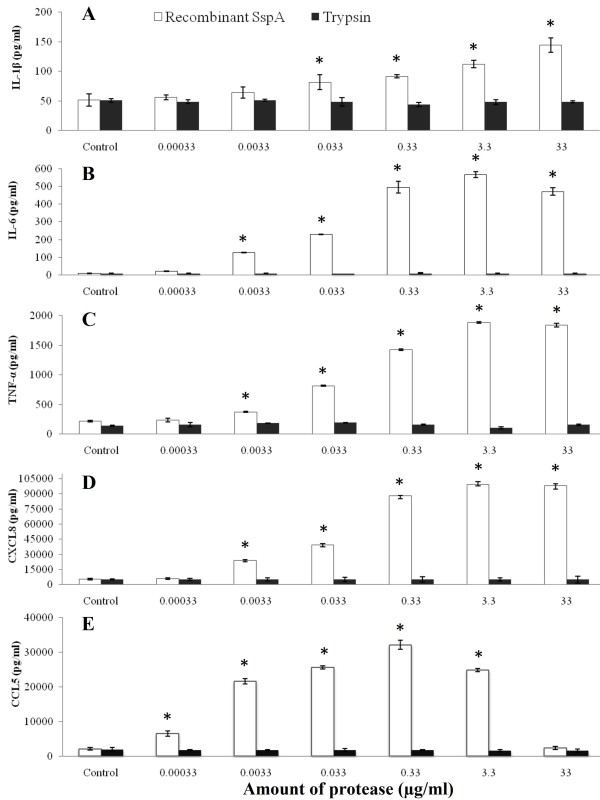
**Cytokine secretion by PMA-differentiated U937 macrophages stimulated with the recombinant SspA of *S. suis *or with pancreatic trypsin**. Following stimulation (18 h) with various amounts of proteases, the secretion of IL-1β (panel A), IL-6 (panel B), TNF-α (panel C), CXCL8 (panel D) and CCL5 (panel E) was assessed by ELISA. The data are the means ± SD of triplicate assays from three separate experiments. Asterisks indicate a significant difference in comparison with the non-stimulated macrophages at P < 0.01.

Prior to determine the capacity of the recombinant SspA of *S. suis *to induce an inflammatory response in PMA-differentiated U937 macrophages, its effect on cell viability was evaluated. The MTT test revealed that macrophage viability was not significantly reduced (less than 20%) by a treatment with the recombinant SspA at a concentration of up to 33 μg/ml. As reported in Figure 1A-C, a significant dose-dependent secretion of all three pro-inflammatory cytokines IL-1β, IL-6 and TNF-α was observed following stimulation of macrophages with the recombinant SspA. More specifically, treatment of macrophages with SspA at 0.33 μg/ml resulted in a 2-fold, 55-fold and 7-fold increase of IL-1β, IL-6 and TNF-α levels, respectively. In addition, there was a significant dose-dependent increase of CXCL8 and CCL5 secretion by macrophages stimulated with the recombinant SspA. The levels of CXCL8 (Figure 1D) increased by 17-fold while that of CCL5 (Figure 1E) increased by 15-fold when the recombinant SspA was used at 0.33 μg/ml (Figure 1D-E). In contrast, when the macrophages were stimulated with pancreatic trypsin instead of recombinant SspA, no increase in cytokine secretion was observed (Figure 1). When macrophages were stimulated with the recombinant SspA at the highest concentration (33 μg/ml), a very low amount of CCL5, which correspond to that of non-stimulated macrophages was detected. This decrease in cytokine production was also observed for IL-6 but to a much lesser extent (Figure 1B).

The effect of stimulating macrophages with heat-inactivated recombinant SspA or with active SspA in the presence of polymyxin (LPS neutralizing molecule) on the secretion of IL-6, CXCL8 and CCL5, the three cytokines produced in higher amounts by macrophages, was then tested. As reported in Table 1, the secretion of IL-6 and CXCL8 was significantly increased after stimulation of macrophages with the active recombinant SspA (33 μg/ml) while only a slight increase was observed in the case of CCL5. The amounts of IL-6 and CXCL8 produced by macrophages were not markedly different when the recombinant SspA of *S. suis *was inactivated by heat treatment (30 min at 100°C). However, stimulation of macrophages with the heat-inactivated SspA was associated with a significantly higher amount of CCL5 in the conditioned culture medium compared to the treatment with the active recombinant SspA (72409 ± 848 versus 2370 ± 61 pg/ml). Lastly, the presence of polymyxin B during stimulation of macrophages with the recombinant SspA protease had no significant effect on the levels of cytokine produced. The efficacy of polymyxin B (1 μg/ml) in neutralizing the inflammatory activity of *Escherichia coli *LPS was demonstrated in preliminary assays.

**Table 1:** Effect of heat treatment or the presence of polymyxin B on cytokine secretion by PMA-differentiated U937 macrophages stimulated with the recombinant SspA (33 μg/ml) of *S. suis*.

Conditions	Amount secreted (pg/ml)		
	CCL5	IL-6	CXCL8
Control (no stimulation)	2081 ± 14	100 ± 1	3170 ± 9
Recombinant SspA of *S. suis*	2370 ± 61*	1922 ± 31*	108557 ± 620*
Heat-inactivated recombinant SspA of *S. suis*	72409 ± 848*	2111 ± 71*	102287 ± 1062*
Recombinant SspA of *S. suis *+ polymyxin B	2081 ± 32	2099 ± 254*	107446 ± 590*

The data are the means ± SD of triplicate assays for three separate experiments. Asterisks indicate a significant difference in comparison with the unstimulated control at P < 0.01.

To further support the inflammatory property of the recombinant SspA, we compared the SspA-deficient mutant G6G and the parental strain for their capacity to induce of IL-1β, TNF-α, IL-6, CXCL8 and CCL5 secretion in macrophages. The MTT test revealed that macrophage viability was not significantly reduced (less than 10%) by a treatment with cells of *S. suis *P1/7 or G6G at MOI of 100. As reported in Table 2, the amounts of IL-1β, TNF-α and IL-6 secreted by macrophages were significantly lower for the SspA-deficient mutant compared to the parental strain. More specifically, IL-1β, TNF-α and IL-6 production were decreased by 26%, 43% and 41%, respectively. In contrast, the amounts of CCL5 and to a lesser extent CXCL8 were significantly higher when macrophages were stimulated with SspA-deficient mutant (G6G) compared to the parental strain.

**Table 2:** Cytokine secretion by PMA-differentiated U937 macrophages following stimulation with *S. suis *P1/7 and its SspA deficient mutant G6G.

Strain	Amount secreted of cytokines (pg/ml)				
	IL-1β	TNF-α	IL-6	CXCL8	CCL5
Control	51 ± 3	217 ± 2	10 ± 1	5245 ± 432	2116 ± 4
*S. suis *P1/7	161 ± 8	1800 ± 11	1160 ± 21	611000 ± 756	13355 ± 564
*S. suis *G6G	120 ± 3*	1030 ± 14*	690 ± 6*	653000 ± 634*	15664 ± 34*

The data are the means ± SD of triplicate assays for three separate experiments. Asterisks indicate a significant difference in cytokine secretion by macrophages stimulated with the SspA deficient mutant (G6G) in comparison with the parental strain at P < 0.01.

**Figure 2:**
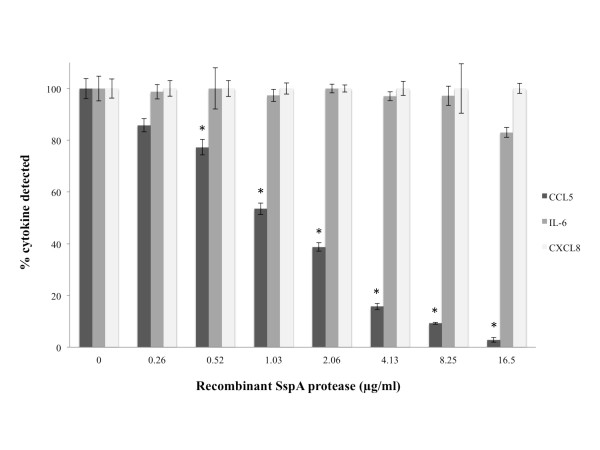
**CCL5, IL-6 and CXCL8 degradation by the recombinant SspA of *S. suis***. A value of 100% was assigned to the amounts of cytokines detected in the absence of SspA. The data are means ± SD of triplicate assays from three separate experiments. Asterisks indicate a significant difference in comparison with the control (no SspA) at P < 0.01.

Lastly we investigated the capacity of the SspA protease to degrade CCL5, IL-6 and CXCL8, the tree cytokines produced in higher amounts by macrophages stimulated with the recombinant SspA. Recombinant cytokines were incubated with the SspA protease at concentrations ranging from 0.26 to 16.5 μg/ml and after 4 h, residual cytokines were determined by ELISA (Figure 2). There was a significant decrease in amounts of CCL5 in presence of SspA, even at low concentrations (0.26 μg/ml). Moreover, a decrease of approximately 20% was also noticed for IL-6 treated with SspA at 16.5 μg/ml. In contrast, there was no decrease for CXCL8 following incubation with SspA.

**Figure 3:**
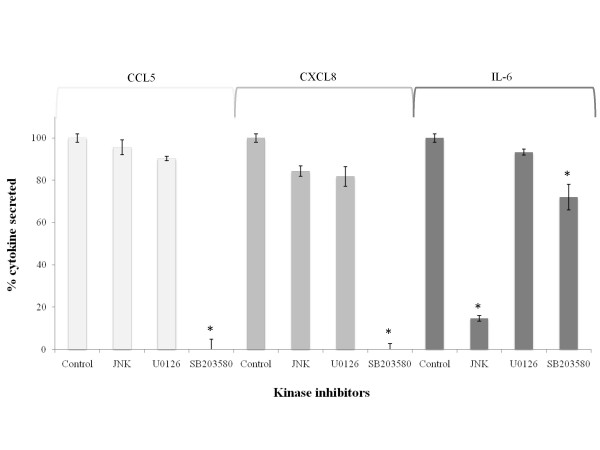
**Effect of kinase inhibitors on the secretion of CCL5, CXCL8 and IL-6 by PMA-differentiated U937 macrophages stimulated with the recombinant SspA (33 μg/ml) of *S. suis***. A value of 100% was assigned to the amounts of cytokines detected in the absence of kinase inhibitors. The data are the means ± SD of triplicate assays from three separate experiments. Asterisks indicate a significant difference in comparison with the control (no inhibitor) at P < 0.01. The JNK inhibitor is specific for c-JUN N-terminal kinase (JNK) inhibitor, U0126 is specific for mitogen-activated extracellular kinase 1, 2 (MEK 1, 2) inhibitor, and SB203580 is specific for p38 mitogen-activated kinase (p38 MAPK) inhibitor.

Thereafter, in order to identify the mechanism by which the recombinant SspA may activate macrophages, the effect of selected kinase inhibitors on the secretion of IL-6, CXCL8 and CCL5 by macrophages was investigated. As reported in Figure 3, a complete inhibition of CCL5 and CXCL8 secretion was observed in the presence of SB203580, an inhibitor specific to p38 mitogen-activated kinase (p38 MAPK). The secretion of IL-6 by this kinase inhibitor was decreased by 28% while it was decreased by 85% with the JNK inhibitor.

## Discussion

*S. suis *is a swine pathogen responsible for several infections including meningitidis, endocarditis and septicemiae, and is also an important agent for zoonosis [1]. Recently, a subtilisin-like protease, named SspA, was identified as a virulence factor in *S. suis*. This was based on the fact that SspA deficient mutants were significantly less pathogenic in animal models [16,17]. In the present study, we sought to determine the capacity of *S. suis *SspA to induce an inflammatory response in U937 macrophages.

We showed that recombinant SspA induced the secretion of IL-1β, TNF-α, IL-6, CXCL8 and CCL5 by macrophages. This significant cytokine secretion may be of utmost importance in *S. suis*-induced meningitis. Indeed, Lopes-Cortes et al., demonstrated that IL-1β and TNF-α are present in the cerebrospinal fluid and that high levels of these cytokines correlate with the neurological complications [25]. More specifically, IL1-β can enhance the permeability of the blood-brain barrier [26]. Moreover, high levels in local body fluids and in serum of IL-6 and TNF-α are associated with a fatal outcome [27]. Moller et al., also reported that the cerebrospinal fluid of patients suffering from bacterial meningitis contains much higher levels of chemokines, including CXCL8 [28].

To ensure that cytokine secretion by SspA-stimulated macrophages did not result from LPS contaminants, polymyxin B, an LPS-reacting molecule [29], was included durind stimulation. Results showed that polymyxin B, did not inhibit cytokine secretion thus suggesting that this stimulation is induced by the recombinant SspA protease only. This ability of the recombinant SspA to induced cytokine secretion in macrophages was found to be highly specific since it was not observed with the pancreatic trypsin used as a control.

Proteases can induce the secretion of inflammatory mediators in mammalian cells by two ways: action on proteinase-activated receptors (PARs) or through a non-proteolytic mechanism, involving the mitogen-activated protein kinases (MAPK) [30,31]. Several proteases have been identified as signaling molecules that specifically regulate members of PARs, a family of seven transmembrane domains G-protein-coupled receptors [32,33]. This family includes four members: PAR-1, PAR-3 and PAR-4 are receptors for thrombin, trypsin or cathepsin G, while PAR-2 is resistant to thrombin, but can be activated by trypsin, mast cell tryptase [30,34-36]. Since the heat-inactivated SspA still possessed the capacity to induce cytokine secretion in macrophages, the involvement of PARs could be ruled out. We thus investigated whether the SspA may induce cytokine secretion through activation of MAP kinases. More specifically, there are three major groups of MAPK in mammalian cells: the extracellular signal-regulated protein kinase (ERK), the p38 MAPK and the c-Jun NH2-terminal kinase (JNK) [31]. Our results obtained by including kinase inhibitor during stimulation of macrophages with the recombinant SspA suggested that the production of CCL5 and CXCL8 was regulated by p38 MAPK while the production of IL-6 was mostly regulated by JNK. MAPK are known as key regulators for the synthesis of numerous cytokines, chemokines, and other inflammatory mediators [31]. Previous studies also suggested a similar involvement of the MAPK regulatory pathway in inflammatory responses induced by *S. suis *[37-39]. In agreement with our observations, the cysteine proteinases of *Porphyromonas gingivalis *was also reported to use the MAPK transduction pathway to induce cytokine secretion in macrophages [40] and fibroblasts [41].

Our data showed that the amounts of CCL5 in the conditioned medium of macrophages stimulated with the heat-inactivated recombinant SspA was higher compared to that detected following stimulation with the active SspA. This suggests that SspA may degrade this cytokine. Using ELISA, we clearly showed the capacity of the recombinant SspA to degrade dose-dependently CCL5. Since CCL5 possesses chemotactic activity for immune cells, its inactivation by the SspA may allow *S. suis *to avoid and delay neutrophil attraction and activation. Cytokine degradation by proteases is a phenomenon well described in group A streptococci. Sumby et al., reported the ability of *Streptococcus pyogenes *SpyCEP to reduce neutrophil activity though cleavage and inactivation of the human chemokine granulocyte chemotactic protein 2 (GCP-2) [42]. In addition, the protease of *S. pyogenes *was reported to cleave CXCL8 [42,43]. Moreover, Bryan et al., showed that *Streptococcus agalactiae *CspA, inactivates the CXC chemokines GRO-alpha, GRO-beta, GRO-gamma, neutrophil-activating peptide 2 (NAP-2), and GCP-2 [44]. Lastly, the subtilisin-like protease SufA of *Finegoldia magna*, that shares many properties with the SspA of *S. suis*, has been shown to degrade the chemokine MIG/CXCL9 [45]. Degradation of CXCL8 by *S. suis *has been previously reported [46]. The protease involved in the cleavage of CXCL8 was different from the cell wall-anchored SspA since it was found to be secreted by *S. suis *[46].

The ability of SspA to induce cytokine secretion in macrophages was confirmed using a mutant of *S. suis *deficient in SspA expression. The secretion of IL-1β, TNF-α, and IL-6 was significantly less important when macrophages were stimulated with cells of SspA mutant compared to the stimulation with the parental strain. This strongly supports the contribution of SspA in *S. suis *induced inflammatory response in macrophages. On the other hand, CCL5 secretion was found to be higher following stimulation with the SspA-deficient mutant compared to the parental strain. This result supports the capacity of the recombinant SspA protease to degrade CCL5. The fact that no decrease in CXCL8 secretion was observed following stimulation of macrophages with the SspA-deficient mutant suggests that other cell surface components of *S. suis*, such as the cell wall [46], are likely to play a more important role in CXCL8 secretion than the SspA protease.

## Conclusions

In conclusion, this study bought evidence that the subtilisin-like protease SspA of *S. suis *may modulate the inflammation state associated with meningitis. It may either induce the secretion of important pro-inflammatory cytokines or, when present at high concentration, cause the degradation of selected cytokines, such as CCL5 and IL-6.

## Authors' contributions

LB performed all the experimental work and prepared the first draft of the manuscript. DG conceived the study design and prepared the final version of the manuscript. All authors read and approved the final manuscript.
